# Comparative Analysis of the Influence of Mineral Engine Oil on the Mechanical Parameters of FDM 3D-Printed PLA, PLA+CF, PETG, and PETG+CF Materials

**DOI:** 10.3390/ma16186342

**Published:** 2023-09-21

**Authors:** Elvis Hozdić, Emine Hozdić

**Affiliations:** 1 Faculty of Mechanical Engineering, University of Novo Mesto, Na Loko 2, 8000 Novo Mesto, Slovenia; 2 Kranj School Centre, Kidričeva Cesta 55, 4000 Kranj, Slovenia; emine.hozdic@sckr.si

**Keywords:** FDM, mechanical properties, mineral engine oil, PLA, PLA+CF, PETG, PETG+CF

## Abstract

Polymer materials and composites play a pivotal role in modern industry, prized for their durability, light weight, and resistance to corrosion. This study delves into the effects of mineral engine oil exposure on the mechanical parameters of 3D-printed materials created through fused deposition modeling (FDM). The research scrutinizes prototype materials under diverse environmental conditions, with a particular focus on the tensile parameters. The primary aim is to analyze and compare how mineral engine oil affects the mechanical parameters of four commonly used FDM 3D-printed materials: PLA, PLA+CF composites, PETG, and PETG+CF composites. In the case of the PLA specimens, the tensile strength decreased by approximately 36%, which, considering the 30% infill, remained acceptable. Simultaneously, the nominal strain at the point of breaking increased by 60.92% after 7 days and 47.49% after 30 days, indicating enhanced ductility. Interestingly, the PLA’s Young’s modulus remained unaffected by the oil. The 3D-printed PLA+CF materials exposed to 30 days of mineral engine oil displayed a substantial Young’s modulus increase of over 49.93%. The PETG specimens exhibited intriguing behavior, with a tensile strength decrease of 16.66% after 7 days and 16.85% after 30 days, together with a notable increase in the nominal strain at breaking by 21.34% for 7 days and 14.51% for 30 days, signifying enhanced ductility. In PETG material specimens, the Young’s modulus increased by 55.08% after 7 days and 66.27% after 30 days. The PETG+CF samples initially exhibited increases in tensile strength (1.78%) and nominal strain at breaking (6.08%) after 7 days, but later experienced an 11.75% reduction in the tensile strength after 30 days. This research underscores the critical role of material selection in oil-exposed environments and suggests avenues for future exploration, encompassing microstructural analysis, the long-term impact of oil exposure, and broader considerations related to environmental and oil-specific factors. It contributes to a deeper understanding of the intricate interactions between polymer materials and mineral engine oil, offering valuable insights that can enhance industrial applications.

## 1. Introduction

The widespread adoption of additive manufacturing (AM) [1,2,3,4], also known as 3D printing, has revolutionized the manufacturing industry, offering unique advantages such as rapid prototyping, mass customization, personalization, and material diversity. The applications of 3D printing are diverse and continually expanding. According to [5], 3D printing has become a popular manufacturing method in various industries, including aerospace, automobile, biomedical, defense, and dental [6,7].

The International Committee F42 of ASTM International provides a comprehensive definition of additive manufacturing, i.e., additive manufacturing is a transformative process that involves the fusion of materials to fabricate objects directly from 3D computer models. This innovative technique operates on a layer-by-layer basis, standing in stark contrast to traditional subtractive manufacturing methods [4,8].

Based on the chosen material and the method of manufacturing the final product, there is a wide range of additive manufacturing techniques available today [9,10], such as Fused Deposition Modelling (FDM), Selective Laser Sintering (SLS), Stereolithography (SLA), Powder-Bed Fusion (PBF), Laminated Object Manufacturing (LOM), 3D Plotting, Direct Energy Deposition (DED), InkJet Printing (IJP), the Direct Write (DW) technique, etc.

FDM is one of the most prevalent and accessible additive manufacturing techniques for 3D printing [5,11,12,13]. In this technique, thermoplastic filaments are melted and de-posited layer by layer to form the final product. FDM is suitable for a wide range of materials, including acrylonitrile butadiene styrene (ABS), acrylonitrile styrene acrylate (ASA), polyamide (PA), polylactide (PLA), and polyethylene terephthalate glycol-modified (PETG).

Among the various materials used in FDM 3D printing, PLA and PETG have gained popularity due to their favorable mechanical properties and ease of use. To further enhance their performance, reinforcing additives such as carbon fibers (CFs) have been incorporated into these materials, resulting in composites with a superior mechanical strength and stiffness.

Based on a review of the scientific literature, it is possible to conclude that the re-search so far in the field of mechanical properties of 3D-printed materials (tensile, flexural, and torsional) has gone in four main directions: (1) the influence of the input material’s characteristics (type, color, etc.) [14,15,16], (2) the impact of filling design on the mechanical properties [17,18,19,20,21,22,23,24,25], (3) the influence of production process parameters (layer height, air gap, raster width, raster angle, build orientation, number of contours, flour/roof thickness, deposition speed, etc.) [26,27,28,29,30,31,32,33], and (4) the influence of environmental factors (temperature, vibrations, humidity, etc.) [34,35,36].

The Interaction between the 3D-printed parts and the environment can impact on their mechanical properties, potentially compromising the structural integrity and performance of the printed components. Therefore, a thorough understanding of the influence of environmental aspects on different 3D-printed materials is crucial for their reliable and efficient utilization in practical applications. In real-world applications, 3D-printed parts often encounter harsh environments such as environmental temperature [37], environ-mental humidity [11,35,38,39], lubricants such as engine oil, etc. 

The study discussed In reference [39] examined the water absorption behavior of FDM components. Additionally, it quantitatively measured the impact of temperature and humidity on the tensile strength, modulus, and strain. These findings have contributed to the design considerations for FDM parts intended for use in diverse environmental conditions.

Costa et al. discovered that a 20 °C rise in the environmental temperature resulted in a 50% reduction in the structural porosity of 3D-printed acrylonitrile butadiene styrene (ABS) [37].

Regarding the polycarbonate (PC) of our interest, several decades ago, there were studies on water absorption that examined alterations in the physical properties [40] and their impact on injection molding processes [41]. Numerous studies have shown that the mechanical characteristics of PC diminish following the absorption of water [42,43,44].

The study presented in [45] examined how environmental factors, like temperature and humidity, affect carbon-fiber-reinforced plastic (CFRP) composites made using AM. It assessed three environmental conditions, i.e., warm and wet, warm and dry, and cold and dry, in comparison to samples tested in standard conditions right after fabrication. The results showed that warm temperatures had a minimal impact on the mechanical performance, while near-zero-degree cold temperatures affected it after 96 and 250 h of exposure. Temperature was a more significant factor in influencing the mechanical properties than humidity.

The behavior of products created with 3D printing technology are presented in [46]. The authors’ findings pertain to the impact of the degradation factor on the mechanical properties of PLA samples and their potential practical applications, particularly in technology. 

The paper in [47] outlines an assessment of the mechanical and physical properties of the tested materials both before and after chemical exposure. In [47], it was revealed that the chemical compounds (acetone, U6002 solvent, ethanol) responsible for the most substantial weight increase in the tested samples were also the primary drivers of significant degradation in the test specimens.

Based on an analysis of the scientific literature addressing the mechanical properties of 3D-printed materials [14,15,16,17,18,19,20,21,22,23,24,25,26,27,28,29,30,31,32,33,34,35,36], it is possible to conclude that the most used international standards for testing materials manufactured through polymer injection processes are ISO 527-2 [48] and ASTM D638-14 [49].

One of the key factors influencing the mechanical parameters and properties of polymeric materials and their composites is their microstructure. Microstructural defects, such as microcracks and porosity, can have a significant impact on the strength, stiffness, ductility, and resistance of materials.

Understanding the mechanism by which mineral engine oil affects the microstructural defects in polymeric and composite materials is crucial for the development of better materials and the improvement of their mechanical parameters and properties.

Following an extensive review of the scientific literature, no systematic study was discovered that investigates the impact of mineral engine oil on the mechanical parameters and properties of FDM 3D-printed polymer materials and composites. This circumstance sets the stage for the current research. 

Mineral engine oil, such as SAE 15W-40/API SF/CD, contains a complex mixture of chemicals that can behave differently in various situations. Polymeric materials and their composites can absorb certain components of mineral engine oil. This absorption depends on the chemical composition of the polymeric material and the oil, as well as environmental conditions such as temperature and pressure. Once the oil is absorbed, it begins to affect the microstructural properties of the polymeric material. Absorbed molecules of mineral engine oil can trigger the formation of microcracks and other microstructural defects in materials. One potential pathway is that the oil promotes the weakening of bonds between the polymeric chains of the material, which can lead to a reduced strength and an increased brittleness of the material. Additionally, the interaction between the oil and the material can also induce chemical reactions that further compromise the microstructure.

The influence of mineral engine oil on microstructural defects in polymer materials can lead to significant alterations in the mechanical parameters of these materials. For instance, materials exposed to oil may exhibit reduced strength, elasticity, and ductility. This has crucial implications for various industries that employ these materials, as such changes can impact the safety and durability of the final products.

The aim of this study was to investigate and compare the effects of mineral engine oil (SAI 15W-40/API SF/CD) on the mechanical parameters of four commonly used FDM 3D-printed materials: PLA, PLA+CF composites, PETG, and PETG+CF composites. By systematically studying these materials under controlled experimental conditions, we gained an insight into their response to lubricant (mineral engine oil) exposure in terms of the mechanical parameters, including the modulus of elasticity (Young’s modulus), the time to the break, the tensile strength, and the nominal strain at the break. These properties are critical indicators of a material’s structural integrity and performance under varying loading conditions. By evaluating the changes in these parameters before and after exposure to engine oil, we can assess the material’s resistance to degradation, the potential loss of mechanical performance, and any possible differences between the pure materials and their reinforced counterparts. The primary mechanism behind these changes was attributed to microstructural defects, as absorbed molecules of mineral engine oil can induce the formation of microcracks.

The obtained results of this research expand the knowledge about the Influence of mineral engine oil on 3D-printed materials. In the future, these findings will aid in the se-lection of appropriate materials for specific environments exposed to lubricants, allowing engineers and designers to make informed decisions and optimize the performance and reliability of 3D-printed components.

The structure of the paper is as follows. Section 2 provides detailed descriptions of the materials used, including PLA, PLA+CF composites, PETG, and PETG+CF composites, and the utilization of the FDM technique of AM, which enables the production of 3D-printed tensile-testing specimens. The experimental results are presented in Section 3. Finally, Section 4 concludes the work by summarizing the research and offering suggestions for future investigations. 

## 2. Materials, Methods, and Equipment

### 2.1. Material Specification for 3D Printing 

Four 3D printing material types were used: PLA, PLA+CF, PETG, and PETG+CF. PLA is a bio-degradable, bio-based thermoplastic polymer derived from renewable resources such as potato starch or sugar cane [12,13]. It has gained popularity in 3D printing due to its ease of use, wide availability, and favorable mechanical properties. PLA exhibits excellent printability and dimensional accuracy, making it suitable for various applications, ranging from prototyping to functional parts. In terms of mechanical properties, PLA has a relatively low tensile strength compared to other engineering-grade materials [5,50]. The tensile strength, flexural strength, elastic modulus, shear stress, and impact strength of the PLA material are discussed in [12,51,52,53]. However, it offers good stiffness and rigidity, which contributes to its structural integrity. PLA is also characterized by its high impact resistance, allowing it to withstand moderate forces without fracturing or breaking. Additionally, PLA has a relatively low melting point, making it compatible with a wide range of 3D printers [5,54,55].

PLA+CF is a composite material obtained by incorporating carbon fibers into PLA. The addition of carbon fibers enhances the mechanical properties of PLA, resulting in improved strength, stiffness, and thermal stability. Cas are known for their high tensile strength and exceptional stiffness-to-weight ratio, making them ideal reinforcement agents. The presence of CF in PLA+CF composites increases their tensile strength, allowing them to withstand higher loads and forces compared to pure PLA. Furthermore, the stiffness of the material is greatly enhanced, providing improved dimensional stability and resistance to deformation. The thermal stability of PLA+CF composites is also improved, enabling them to withstand higher temperatures without significant degradation. The mechanical properties of PLA+CF composites are discussed in [56,57,58].

PETG is a thermoplastic co-polyester that offers a combination of excellent mechanical properties, chemical resistance, and ease of use in 3D printing. PETG is known for its high strength, durability, and impact resistance, making it suitable for a wide range of applications. It is often chosen for producing functional prototypes, mechanical parts, and end-use products. In terms of mechanical properties, PETG exhibits good tensile strength, allowing it to withstand loads and forces. It also possesses excellent impact resistance, making it highly resistant to cracking or breaking under sudden impacts or stresses. The influence of the process parameters on the mechanical properties of PETG materials has been investigated [59,60]. PETG has low shrinkage during the printing process, resulting in improved dimensional stability and minimal warping. It is also known for its high transparency and clarity, providing visually appealing prints [54,61,62,63].

PETG+CF is a composite material that combines the properties of PETG with the reinforcement of carbon fibers [64,65,66,67]. The incorporation of carbon fibers into PETG enhances its mechanical strength, stiffness, and thermal stability. This composite material is particularly suitable for applications requiring high-performance properties. PETG+CF composites exhibit an improved tensile strength and stiffness compared to pure PETG. The carbon fibers provide exceptional reinforcement, allowing the material to withstand higher loads and forces. Moreover, PETG+CF composites retain the excellent impact resistance of PETG, making them resistant to cracking or breaking under sudden impacts. The thermal stability of PETG+CF composites is also enhanced, enabling them to withstand elevated temperatures without significant degradation.

The specifications and mechanical parameters of PLA, PLA+CF, PETG, and PETG+CF filaments employed according to the manufacturer are presented in Table 1 and Table 2 [68]. The mechanical parameters shown in Table 2 are obtained from the filament manufacturer [68] and relate to the 100% density of the test specimen’s infill reinforcement.

Based on the material specification provided by the filament manufacturer [68], this research used circular cross-section filaments with an average diameter of 1.75 mm and minimal standard deviation.

### 2.2. Preparation and Printing of Tensile Test Specimens

The experimental study involved using the 3D CAD software SolidWorks 2020 for the specimen design, slicer FleshPrint5 software (http://www.flashforge.com/ accessed on 18 August 2023) for adjusting the printer parameters, and the Adventurer 4 Series 3D printer for manufacturing the tensile test specimens.

**Figure 1:**
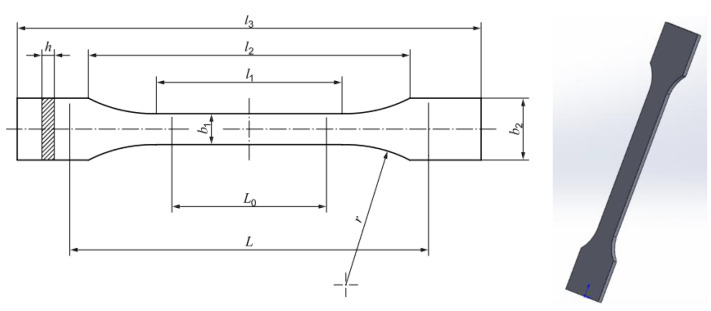
3D model of tensile test specimen according to ISO 527-2 standard.

The tensile test specimen model was prepared in the 3D CAD software SolidWorks 2020 according to the ISO 527-2 standard [48] (Figure 1 and Table 3), and was converted to STL format for the slicer FleshPrint5 software.

The STL file represents the input parameter for the process of setting and adjusting the process parameters for the 3D printing. The main printing parameters for every material are presented in Table 4.

**Figure 2:**
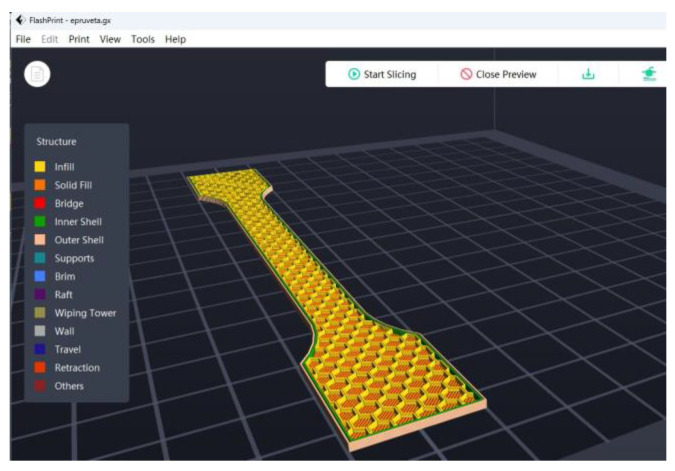
Tensile test specimen with “Hexagon” infill pattern and 30% infill density.

In the slicer FleshPrint5 software, the user has the option to select 4 different infill pat-terns: Line, Hexagon, Triangle, and 3D Infill. For this study, for all the tensile test specimens, the “Hexagon” infill pattern was used with 30% fill density (Figure 2).

After configuring all the parameters in the FlashPrint5 software’s slicer (Table 3), the G-code was generated and transferred to the 3D-printer-controlled computer. Subsequently, all the tensile test specimens were printed using the Adventurer 4 Series 3D printer.

The 3D printer Adventurer 4 Series incorporates a touchscreen display, which not only provides intuitive navigation but also offers comprehensive status updates throughout the printing process. This interface provides swift access to a wide range of essential parameters. These parameters include options such as layer height, print speed, and temperature settings. With just a few simple taps or adjustments, users can easily fine-tune these settings to achieve the optimal print quality and desired outcomes.

For each material, nine identical tensile test specimens were printed, resulting in a total of 36 specimens. All the tensile test specimens were built with 1000 g spools of PLA, PLA+CF, PETG, and PETG+CF materials. The PLA+CF and PETG+CF composite materials were prepared at the filament manufacturer [68].

Table 5 shows the recorded parameters during the 3D printing process of all the tensile test specimens using the Adventurer 4 Series 3D printer.

### 2.3. Tensile Testing for 3D-Printed Specimens

The input for the tensile testing is represented by the 3D-printed tensile test specimens. The 3D-printed tensile test specimens of each material are categorized into three groups and subjected to testing at specific time intervals. Initially, three samples of each material are tested without any exposure to environmental factors. Subsequently, the remaining samples are immersed in a container filled with mineral engine oil (SAI 15W-40/API SF/CD). The second group of samples is tested after 7 days, while the final group of samples for all materials is tested after a 30-day period.

The testing procedure employed in this research is explained according to [48]. The main challenges encountered during the testing of samples made from polymer materials and composites obtained through FDM were as follows: (1) specimen fracture due to stress concentration, (2) software not recording the breaking forces for certain samples, (3) specimen detachment from the machine during testing (Case 4, Case 7, Case 11, Case 12), especially in samples exposed to the influence of mineral engine oil (Case 6, Case 8, Case 9), etc.

**Figure 3:**
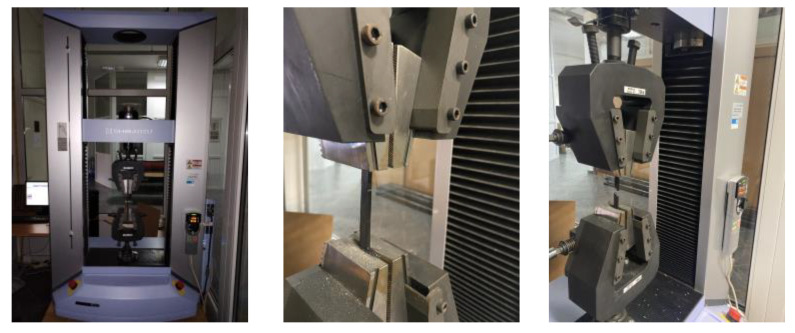
Tensile testing of 3D-printed specimens.

Tensile tests were conducted using a Shimadzu AGS-X universal testing machine (Figure 3) with a maximum load of 10 kN with a testing speed of 6mm/min, according to the ISO 527-2 standard. All the 3D-printed tensile test specimens were statically loaded.

The output values, in turn, consist of the tested specimens and the tensile test data.

The tensile test data acquisition and monitoring were facilitated by Shimadzu Trapezium-X software. This powerful software not only enables the collection of data such as displacement (mm), force (N), and time(s) for each test, but also facilitates the generation of force–displacement curves.

After acquiring the tensile test data, the results were processed using the Excel pro-gram and are presented in the next section. Using the data obtained from the tensile test, the mechanical parameters (tensile strength and nominal strain at the break) of the tensile test specimens were calculated. The tensile strength σ was determined using Equation (1), where F represents the maximum force, and A denotes the cross-sectional area at the narrowest part. The nominal strain at the break ε was computed using Equation (2), where ∆L represents the maximum displacement and L the initial distance between grips; see Figure 1.

(1) $$\sigma =\frac{F_{max}}{A}$$

(2) $$\varepsilon =\frac{∆L}{L}\times 100\%$$

The next section presents the results obtained from testing the tensile mechanical parameters of 3D-printed PLA, PLA+CF, PETG, and PETG+CF tensile-testing specimens.

## 3. Results

### 3.1. Mechanical Properties of the 3D-Printed and Tensile-Tested PLA Specimens

**Figure 4:**
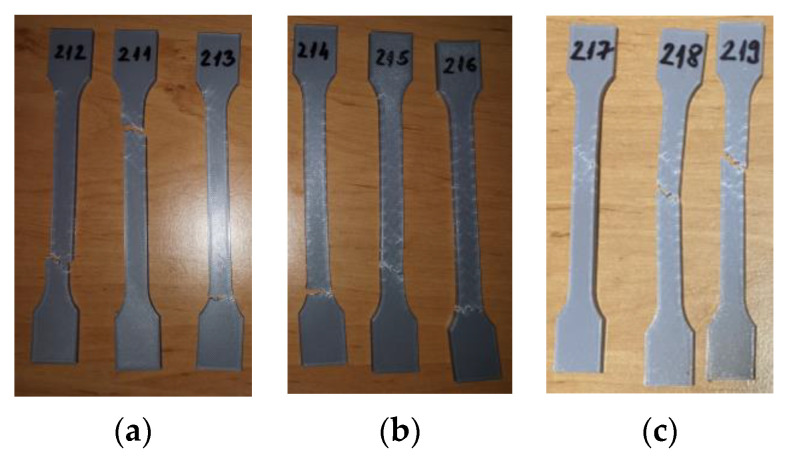
3D-printed PLA specimens tested: (**a**) no mineral engine oil, (**b**) after a 7-day period, (**c**) after a 30-day period.

The 3D-printed PLA tensile-testing specimens after the tensile testing are presented in Figure 4.

The tensile test data for all 3D-printed PLA specimens are shown in Table 6. The data are organized into three distinct groups: (1) data pertaining to tested 3D-printed PLA specimens that were not exposed to mineral engine oil (PLA_211, PLA_212, and PLA_213)—Case 1, (2) data concerning tested 3D-printed PLA specimens that were exposed to mineral oil for 7 days (PLA_214, PLA_215, and PLA_216)—Case 2, and (3) data relating to tested 3D-printed PLA specimens that were exposed to mineral oil for 30 days (PLA_217, PLA_218, and PLA_219)—Case 3.

**Figure 5:**
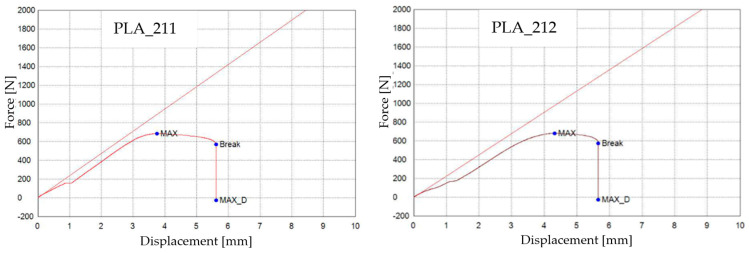

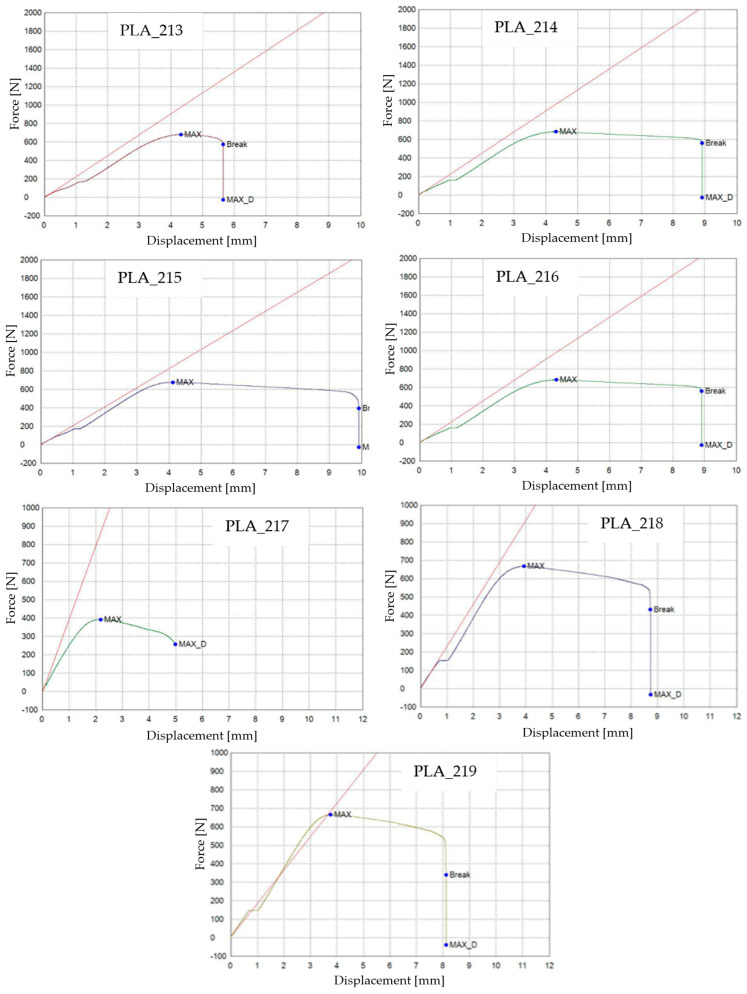
3D-printed PLA specimens tested: the force–displacement curves.

The graphical representations of the maximum force, breaking force, and maximum displacement force for individual PLA specimens are shown in Figure 5.

Based on the analysis of the graphs in Figure 5, it is evident that the 3D-printed PLA_214, PLA_215, and PLA_216 specimens (Case 2) demonstrated a notable increase in displacement (approximately up to 9 mm) when compared to the 3D-printed PLA specimens that were not exposed to mineral engine oil (PLA_211, PLA_212, and PLA_213).

**Figure 6:**
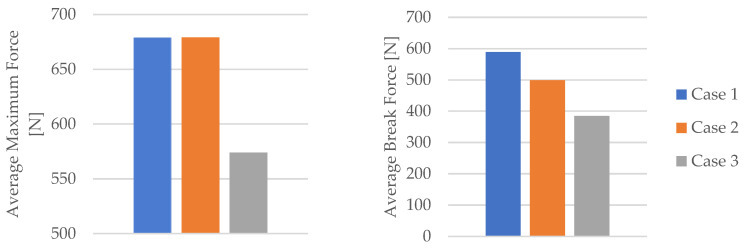
Average maximum force and average break force of the 3D-printed PLA specimens tested.

**Figure 7:**
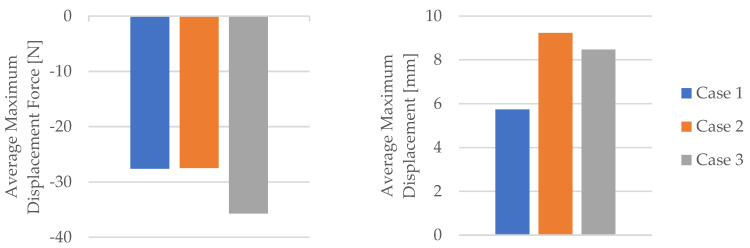
Average maximum displacement force and average maximum displacement of the 3D-printed PLA specimens tested.

The impact of mineral engine oil on the behavior of the 3D-printed PLA specimens is evident in both the average maximum force and the average breaking force, as depicted in Figure 6, as well as the average maximum displacement force and average maximum displacement, as illustrated in Figure 7.

**Figure 8:**
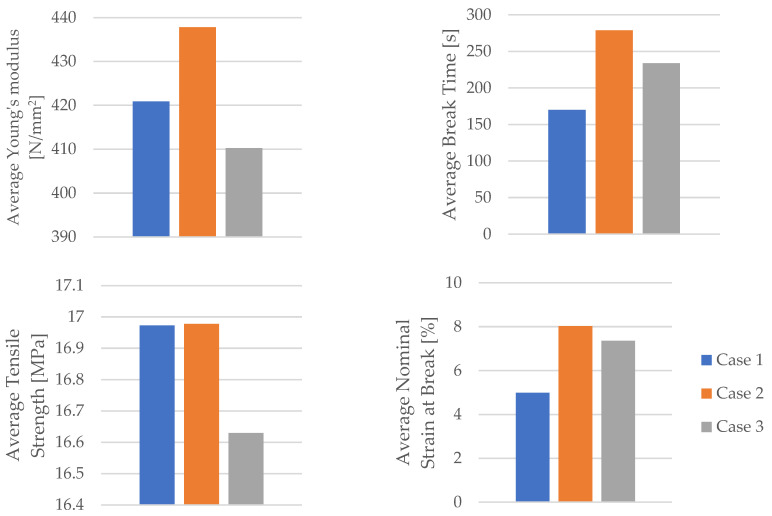
The mechanical parameters of 3D-printed PLA specimens tested.

Using the data obtained from the tensile test in Table 6 as well as Equations (1) and (2), the mechanical parameters (tensile strength, nominal strain at the break) of the PLA tensile test specimens were calculated. Table 7 and Figure 8 display the corresponding average tensile strength and average nominal strain at the break. The average Young’s modulus and average time of the break shown in Table 7 and Figure 8 were recorded during the test.

**Figure 9:**
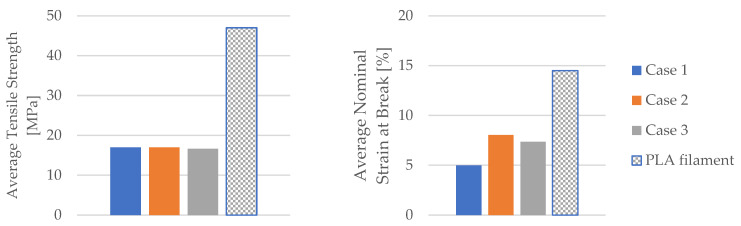
The comparison of the mechanical parameters of PLA tensile-tested specimens with those of the filament.

The displayed mechanical parameters of the tested 3D-printed PLA specimens in Figure 9 (average tensile strength and average nominal strain at the break) refer to the specimens with 30% infill. The manufacturer of the filaments used in this study provided the mechanical parameters of the PLA filament for a 100% infill (Table 2). Figure 9 shows a comparison between the obtained mechanical parameters of the tested 3D-printed PLA specimens and the mechanical parameters specified by the manufacturer of the PLA filament used.

When comparing the average tensile strengths between the manufacturer’s specified values for the PLA filament and the values obtained from testing the 3D-printed PLA specimens, it becomes apparent that the 3D-printed PLA specimens achieve approximately 36% of the prescribed values set by the filament manufacturer. However, since the 3D-printed PLA specimens were created with a 30% infill, this difference in average tensile strengths can be considered minimal. Furthermore, the influence of mineral engine oil on the average tensile strength of the 3D-printed PLA specimens was found to be negligible.

The analysis of the obtained values for average nominal strain at the break in the 3D-printed PLA specimens indicates a notable increase in samples exposed to mineral engine oil, as shown in Figure 9. On the other hand, the 3D-printed PLA specimens with 30% infill that were not exposed to mineral engine oil achieved approximately 34.41% of the prescribed average nominal strain at the break specified by the PLA filament manufacturer.

The 3D-printed PLA specimens exposed to mineral engine oil for 7 days attained approximately 55.28% of the prescribed average nominal strain at the break specified by the PLA filament manufacturer. Likewise, the 3D-printed PLA specimens exposed to mineral engine oil for 30 days achieved approximately 50.76% of the prescribed value specified by the PLA filament manufacturer (Figure 9).

### 3.2. Mechanical Properties of the 3D-Printed and Tensile-Tested PLA+CF Specimens

**Figure 10:**
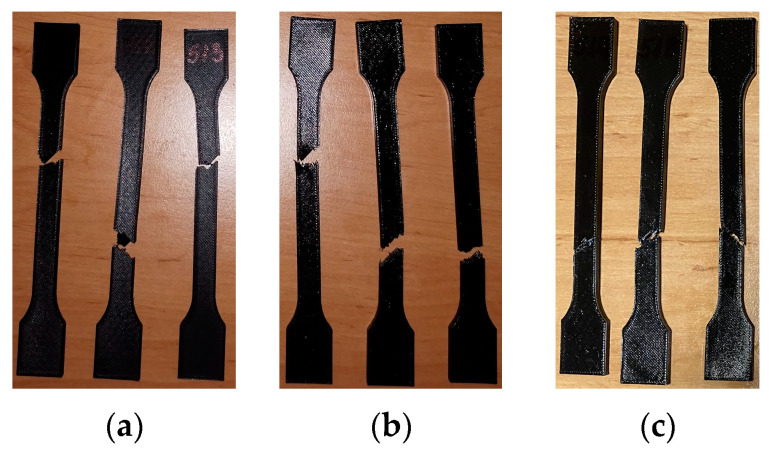
3D-printed PLA+CF specimens tested: (**a**) no mineral engine oil, (**b**) after a 7-day period, (**c**) after a 30-day period.

Figure 10 shows the 3D-printed PLA+CF tensile test specimens after the test. As shown Figure 10, all fractures occur within the distance between broad parallel-sided portions ($l_{2}$).

The tensile test data for all 3D-printed PLA+CF specimens tested are shown in Table 8. The data are organized into three distinct groups: (1) data pertaining to tested 3D-printed PLA+CF specimens that were not exposed to mineral engine oil (PLA+CF_511, PLA+CF_512, and PLA+CF_513)—Case 4, (2) data concerning tested 3D-printed PLA+CF specimens that were exposed to mineral oil for a duration of 7 days (PLA+CF_514, PLA+CF_515, and PLA+CF_516)—Case 5, and (3) data relating to tested 3D-printed PLA+CF specimens that were exposed to mineral engine oil for a period of 30 days (PLA+CF_517, PLA+CF_518, and PLA+CF_519)—Case 6.

**Figure 11:**
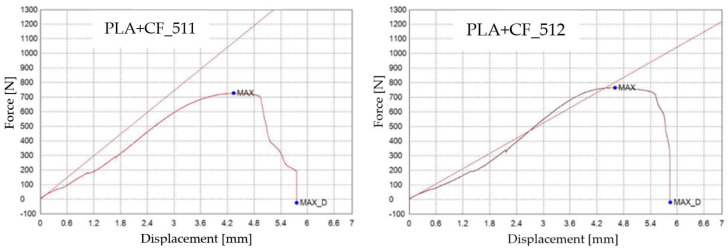

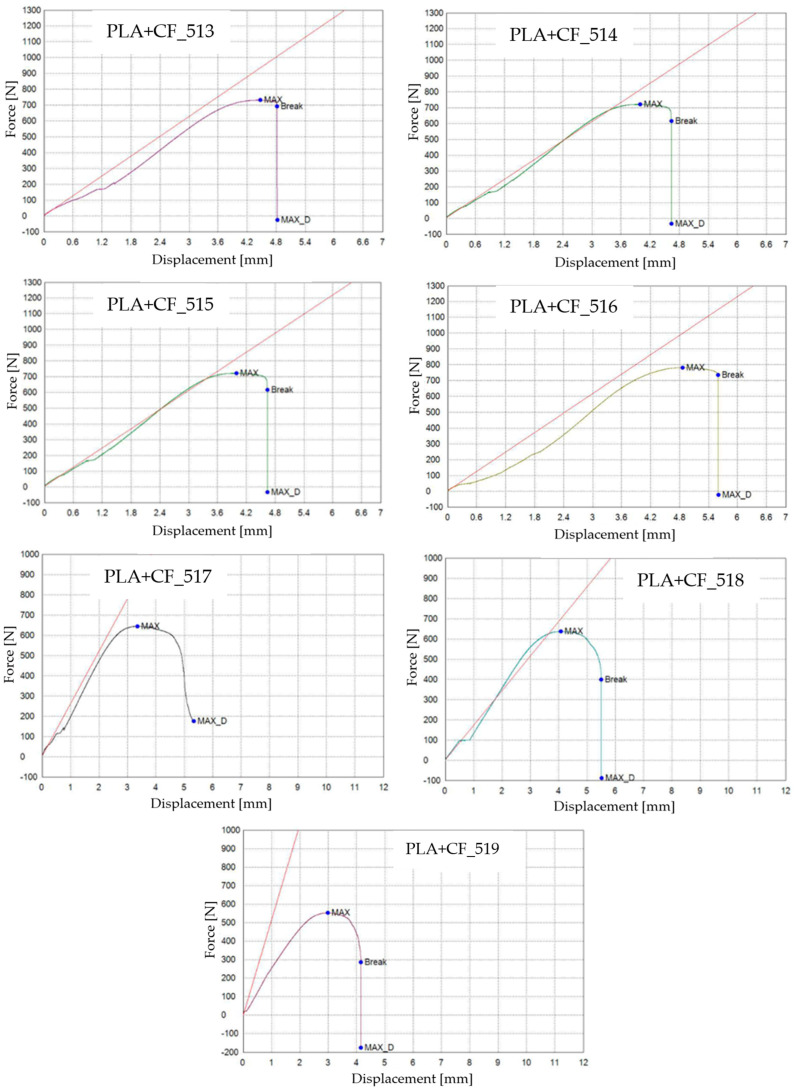
3D-printed PLA+CF specimens tested: the force–displacement curve.

The graphical representations of the maximum force, the breaking force, and the displacement for individual PLA+CF specimens are shown in Figure 11.

**Figure 12:**
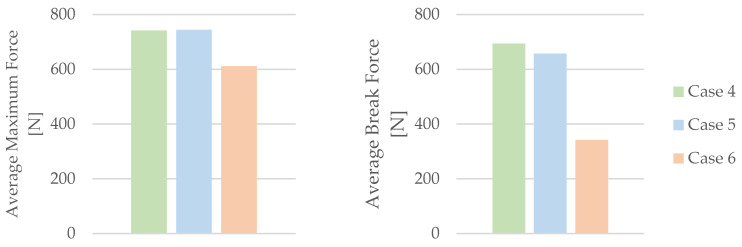
Average maximum force and average break force of the 3D-printed PLA+CF specimens tested.

**Figure 13:**
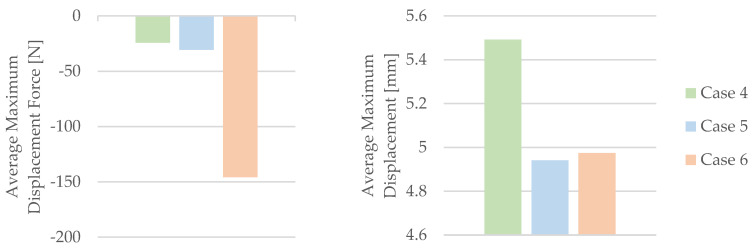
Average maximum displacement force and average maximum displacement of the 3D-printed PLA+CF specimens tested.

The influence of mineral engine oil on the behavior of 3D-printed PLA+CF specimens is also evident in the average maximum force and average breaking force, as shown in Figure 12, and the average maximum displacement force and average maximum dis-placement, as shown in Figure 13.

The analysis presented in Figure 12 indicates a clear trend—the longer the exposure time of the 3D-printed PLA+CF specimens to mineral engine oil, the more pronounced the reduction in both the average maximum force and average breaking force. Moreover, Figure 13 demonstrates a noticeable trend of a decreasing average maximum displacement in the 3D-printed PLA+CF specimens that were subjected to mineral engine oil exposure.

**Figure 14:**
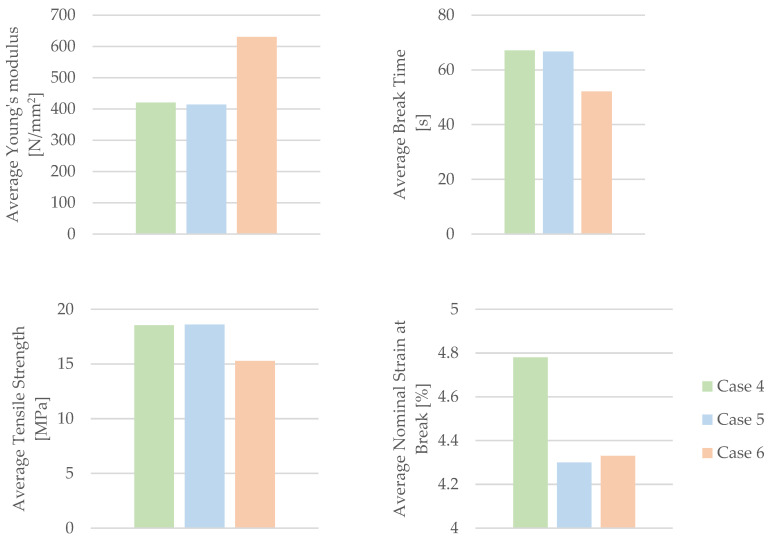
The mechanical parameters of 3D-printed PLA+CF specimens tested.

By utilizing the data acquired from the tensile test outlined in Table 8, the mechanical parameters of the PLA+CF tensile test specimens were computed. Table 9 and Figure 14 present the associated average tensile strength and average nominal strain at the break. The average Young’s modulus and average time of the break, recorded during the test, are also displayed in Table 9 and Figure 14.

Figure 14 clearly demonstrates a trend of diminishing mechanical parameters in the 3D-printed PLA+CF specimens when subjected to the effects of mineral engine oil. This trend is especially prominent in the decreasing values of the average nominal strain at the break (Figure 14).

Based on the information presented in Figure 14, it can be concluded that there was an increase in the Young’s modulus during the testing of PLA+CF specimens that were exposed to mineral engine oil for 30 days.

**Figure 15:**
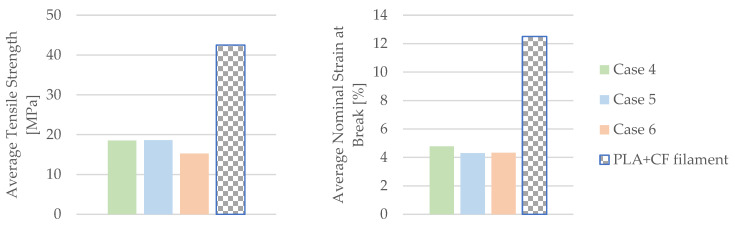
The comparison of the mechanical parameters of PLA+CF tensile-tested specimens with those of the filament.

The results comparing the mechanical parameters of 3D-printed PLA+CF specimens with those of the filament are depicted in Figure 15.

According to the data in Table 2, the manufacturer specifies an average tensile strength of 42.5 MPa and an average nominal strain at the break of 12.5% for the PLA+CF filament. The 3D-printed PLA+CF specimens with 30% infill achieved an average tensile strength of 40.89% of that prescribed by the PLA+CF manufacturer and an average nominal strain at the break of 38.24%. These results are relatively satisfactory, considering the specimens were printed with 30% infill. However, when exposed to mineral engine oil, these values significantly decrease in the 3D-printed PLA+CF specimens.

### 3.3. Mechanical Properties of the 3D-Printed and Tensile-Tested PETG Specimens

**Figure 16:**
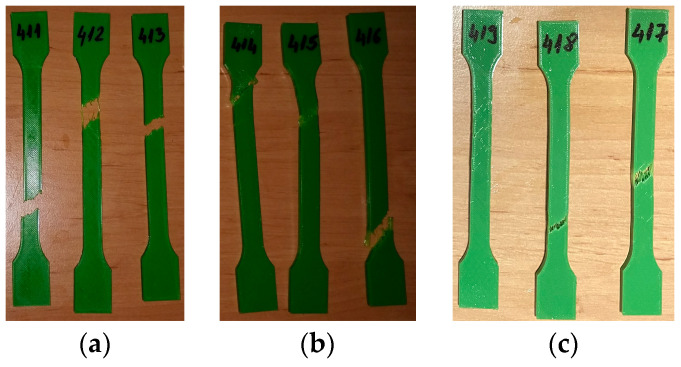
3D-printed PETG specimens tested: (**a**) no mineral engine oil, (**b**) after a 7-day period, (**c**) after a 30-day period.

The 3D-printed PETG tensile test specimens after the test are shown in Figure 16. After testing, the 3D-printed PETG_411, PETG_412, and PETG_413 specimens tested (Figure 16a) displayed a clean and relatively precise fracture within the length of the narrow parallel-sided portion zone (l_2). The remaining 3D-printed PETG specimens, which were subjected to mineral engine oil exposure for 7 days and 30 days, displayed irregular fractures, as illustrated in Figure 16b,c.

**Figure 17:**
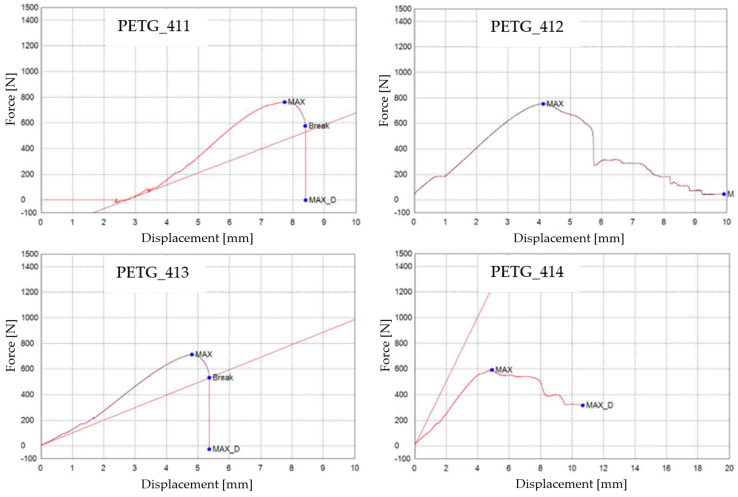

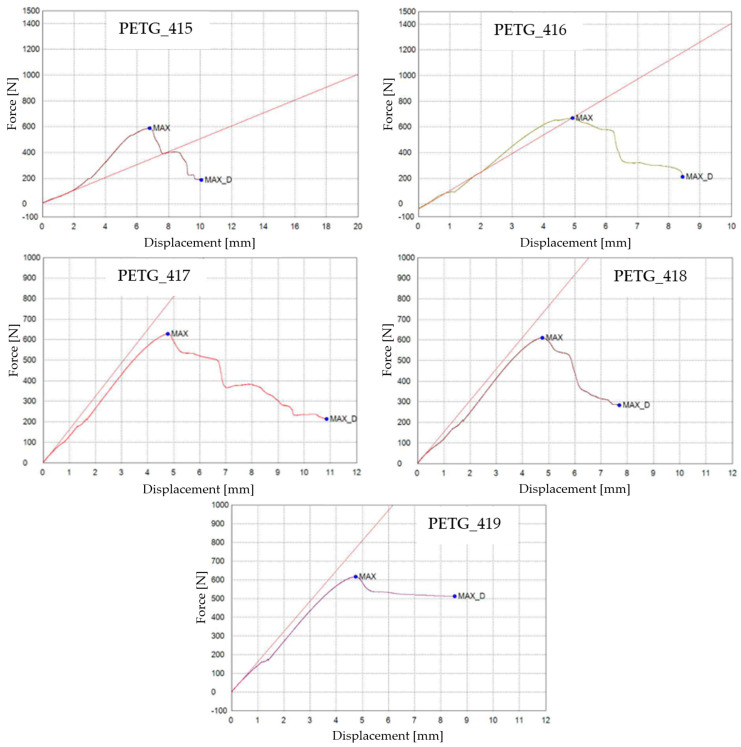
3D-printed PETG specimens tested: the force–displacement curves.

The tensile test data for all 3D-printed PETG specimens are shown in Table 10. The data are organized into three distinct groups: (1) data pertaining to tested 3D-printed PETG specimens that were not exposed to mineral engine oil (Figure 17)—Case 7, (2) data concerning tested 3D-printed PETG specimens that were exposed to mineral oil for 7 days (Figure 17)—Case 8, and (3) data relating to tested 3D-printed PETG specimens that were exposed to mineral oil for 30 days (Figure 17)—Case 9.

As indicated by Table 10, the breaking force data for samples Case 8 and Case 9 were not recorded during the test execution. However, the behavior of the specimens in these cases is indicative and can be observed through the graphs presented in Figure 17. In Figure 17, the graphical representations of the maximum force, the breaking force, and the displacement for individual PETG specimens are shown.

**Figure 18:**
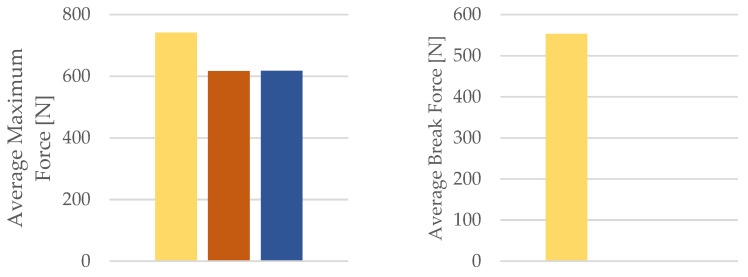
Average maximum force and average break force of the 3D-printed PETG specimens tested.

**Figure 19:**
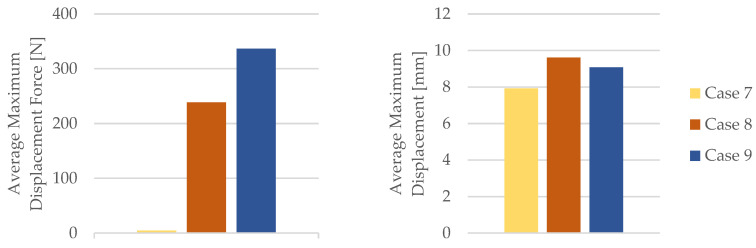
Average maximum displacement force and average maximum displacement of the 3D-printed PETG specimens tested.

The influence of mineral engine oil on the behavior of 3D-printed PETG specimens is also evident from the average maximum force and average breaking force, as shown in Figure 18, and the average maximum displacement force and average maximum dis-placement (Figure 19).

**Figure 20:**
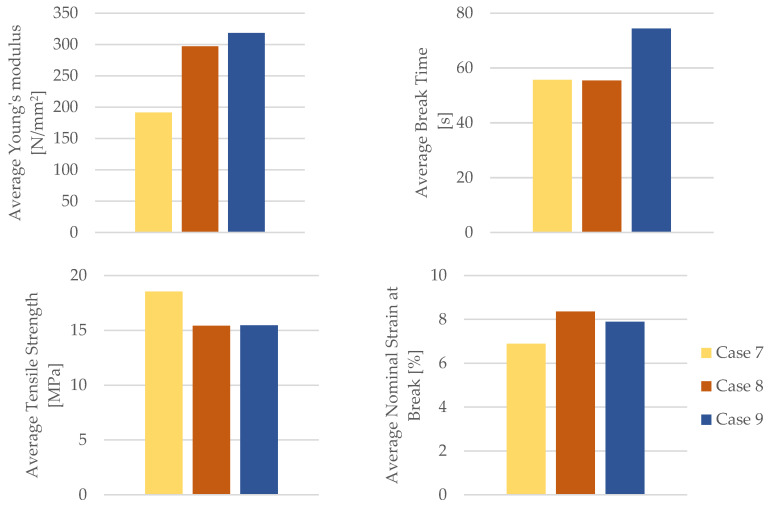
Mechanical parameters of 3D-printed PETG specimens tested.

By employing the data obtained from the tensile test outlined in Table 10, the mechanical properties of the PETG tensile test specimens were calculated. Table 11 and Figure 20 provide the corresponding average tensile strength and average nominal strain at the break.

As evident from Figure 20, the PETG specimens subjected to the influence of engine mineral oil exhibited an increased Young’s modulus, time for the break, and the nominal strain at the break. This effect was more pronounced with a longer exposure to engine mineral oil on the PETG specimens. Simultaneously, due to the action of the engine mineral oil on the PETG material, there was a reduction in the tensile strength.

**Figure 21:**
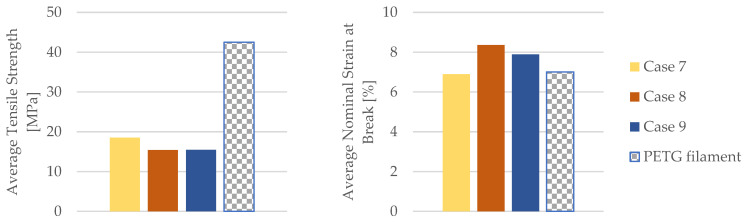
Comparison of the mechanical parameters of PETG tensile-tested specimens with those of the filament.

The results comparing the mechanical parameters of the 3D-printed PETG specimens with those of the PETG filament are depicted in Figure 21.

The insights from Figure 21 underscore a compelling observation—the PETG tensile-tested specimens, when subjected to the effects of mineral engine oil, demonstrated an uptick in the nominal strain at the break, registering an increase of approximately 11%. An intriguing aspect to consider is that these PETG specimens were crafted with a 30% in-fill pattern.

The ramifications of these findings are considerable. The data in Table 11 under-score the advantageous role played by mineral engine oil in enhancing the mechanical parameters of the PETG material. This effect is especially remarkable considering the specimens’ composition with a moderate infill percentage, suggesting that the interaction between the material and the oil has led to a discernible improvement in its ability to withstand strain and deformation. As such, this research presents a compelling case for the beneficial influence of mineral engine oil on the overall performance and durability of the PETG material in load-bearing scenarios.

### 3.4. Mechanical Properties of the 3D-Printed and Tensile-Tested PETG+CF Specimens

**Figure 22:**
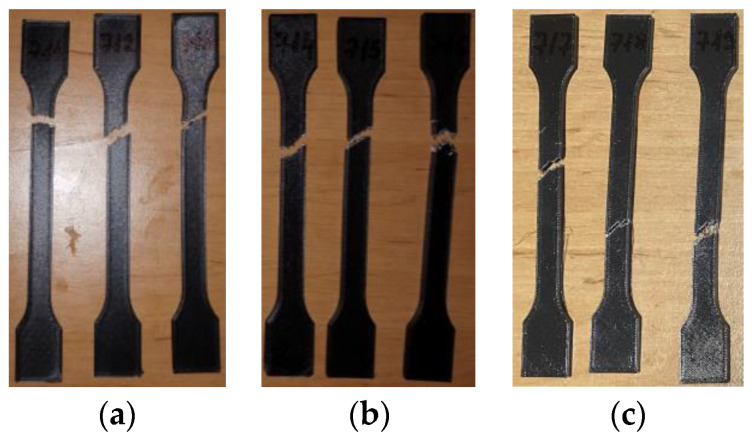
3D-printed PETG+CF specimens tested: (**a**) no mineral engine oil, (**b**) after a 7-day period, (**c**) after a 30-day period.

Figure 22 shows the 3D-printed PETG+CF tensile test specimens after the completion of the test. The specimens are categorized into three groups. The group labelled as “not ex-posed to mineral engine oil” consists of PETG+CF_711, PETG+CF_712, and PETG+CF_713, as depicted in Figure 22a. The group subjected to mineral engine oil exposure for 7 days comprises PETG+CF_714, PETG+CF_715, and PETG+CF_716, as shown in Figure 22b. Finally, the group exposed to mineral engine oil for 30 days includes PETG+CF_717, PETG+CF_718, and PETG+CF_719, as illustrated in Figure 22c.

The tensile test data for all the 3D-printed PETG+CF specimens are shown in Table 12. The data are organized into three distinct groups: (1) data pertaining to tested 3D-printed PETG+CF specimens that were not exposed to mineral engine oil—Case 10, (2) data concerning tested 3D-printed PETG+CF specimens that were exposed to mineral oil for 7 days—Case 11, and (3) data relating to tested 3D-printed PETG+CF specimens that were exposed to mineral oil for 30 days—Case 12.

**Figure 23:**
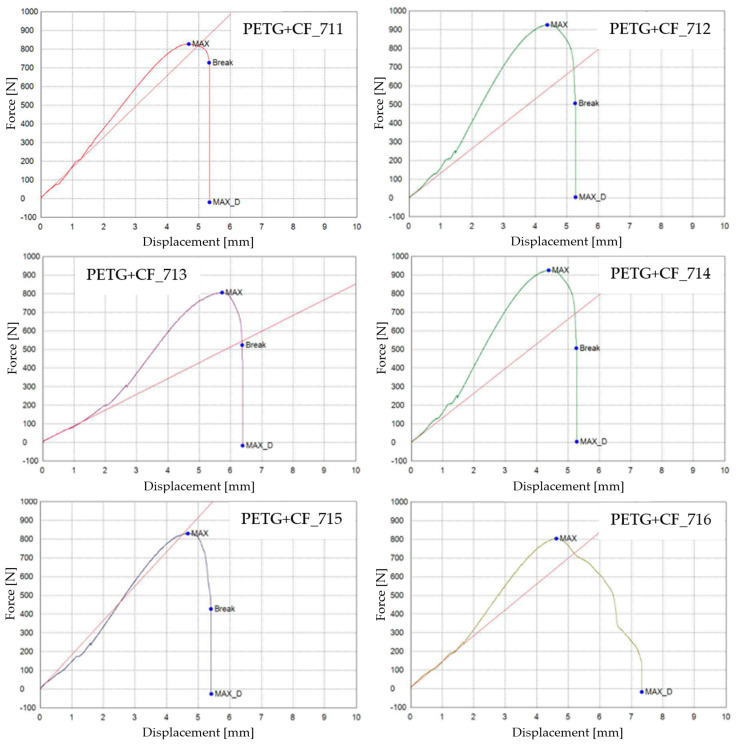

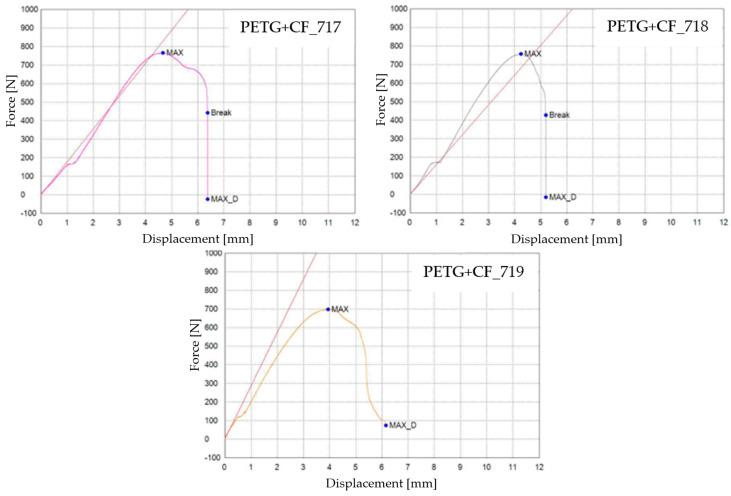
3D-printed PETG+CF specimens tested: the force–displacement curves.

The graphical representations of the maximum force, the breaking force, and the dis-placements for individual PETG+CF specimens are shown in Figure 23.

**Figure 24:**
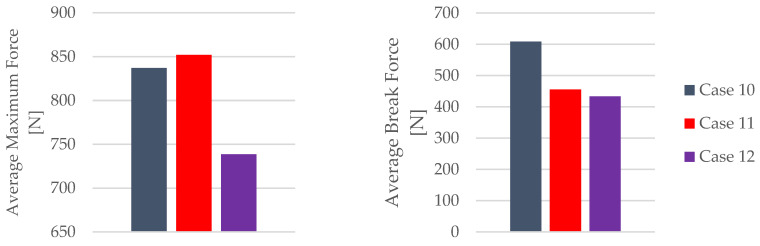
Maximum force and break force of the 3D-printed PETG+CF specimens tested.

**Figure 25:**
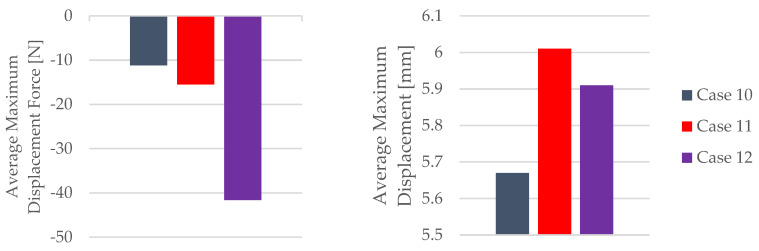
Maximum displacement force and maximum displacement of the 3D-printed PETG+CF specimens tested.

The influence of the mineral engine oil on the behavior of the 3D-printed PETG+CF specimens is also evident in the average maximum force and average breaking force, as shown in Figure 24, and the average maximum displacement force and average maxi-mum displacement (Figure 25).

The insights gleaned from the visualization in Figure 24 provide clear evidence of the effects induced by the presence of the mineral engine oil on the PETG+CF material. Notably, this influence led to a decrease in both the maximum force and the breaking force, indicating an alteration in the material’s mechanical response under the conditions of the exposure; see Table 13. This alteration in force thresholds has implications for the material’s overall strength and integrity.

Conversely, an intriguing and somewhat counterintuitive observation emerges from Figure 25. Despite the reduction in force-related metrics, the application of mineral engine oil correspondingly resulted in an augmentation of the maximum displacement. This augmentation is an important aspect to consider, as it suggests a complex interplay between the mechanical parameters of the PETG+CF material and the oil’s influence. It is plausible that the oil’s presence led to a change in the material’s ability to accommodate deformation without a proportional increase in the applied force, potentially indicating an adaptive response to the oil’s effects.

The combined insights from Figure 24 and Figure 25 unravel a multifaceted interaction between the mineral engine oil and the PETG+CF material. This interaction manifests as a simultaneous reduction in the force thresholds and an increase in the displacement, painting a more comprehensive picture of the material’s behavior under the influence of the mineral engine oil.

**Figure 26:**
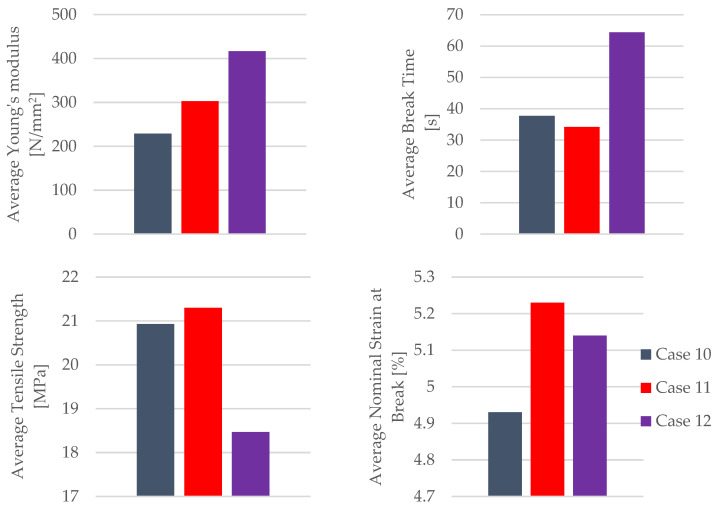
Mechanical parameters of 3D-printed PETG+CF specimens tested.

The mechanical parameters of the 3D-printed PETG+CF specimens tested are presented in Figure 26.

The insights derived from Figure 26 underscore a noteworthy trend—the impact of the mineral engine oil on the PETG+CF material leads to a significant enhancement in both the modulus of elasticity and the time to the break within the PETG+CF specimens. This augmentation suggests a positive adaptation of the material’s mechanical parameters, potentially influenced by the oil’s presence.

However, an intriguing and somewhat counterintuitive pattern emerges when considering the extended exposure to the effects of mineral engine oil on the PETG+CF specimens. This prolonged interaction results in a gradual reduction in both the tensile strength and the nominal strain at the break. This diminishment could be attributed to a variety of factors, including potential degradation or structural alterations induced by the oil’s influence over time.

One particularly intriguing observation arises from the temporal perspective of the material’s response. Initially, with a 7-day exposure to the effects of the mineral engine oil, the PETG+CF exhibits a surge in both the tensile strength and the nominal strain at the break. This phenomenon suggests an early adaptive response of the material to the oil, potentially involving surface modifications or an initial reinforcement effect.

**Figure 27:**
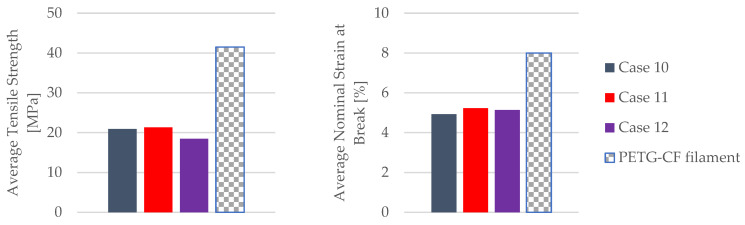
Comparison of the mechanical parameters of PETG+CF tensile-tested specimens with those of the filament.

The results comparing the mechanical parameters of the 3D-printed PETG+CF specimens with those of the PETG+CF filament are depicted in Figure 27.

The insights gleaned from Figure 27 shed light on a compelling trend—PETG+CF tensile-tested specimens exhibit an enhanced nominal strain at the break when subjected to the influence of mineral engine oil. This enhancement is of particular significance, given that these specimens were carefully manufactured with a 30% infill configuration, indicating that the positive effects of the oil are observed within a specific structural context.

Furthermore, an observation emerges from the tensile strengths achieved. These values surpassed the halfway mark, reaching just over 50% of the manufacturer-specified benchmarks for the PETG+CF filament, as meticulously detailed in Table 2.

This elevation in tensile strength suggests that the interaction between the material and the oil, at least initially, led to an augmentation of the material’s capacity to withstand an applied force.

However, it is important to recognize that these amplified values are not permanent. Over time, as the PETG+CF specimens remain exposed to the effects of mineral engine oil, the tensile strengths start to decline. This temporal evolution underscores the complexity of the interplay between the material’s inherent properties and the oil’s impact, suggesting that the initial enhancement might be a transient response or could involve structural alterations that become more prominent with prolonged exposure.

In summary, the findings shown in Figure 27 highlight a nuanced relationship be-tween the mineral engine oil and the PETG+CF material. The enhancements in nominal strain at the break and the tensile strength underscore the material’s adaptability and its potential to respond favorably to external influences.

### 3.5. Comparative Analysis

Below is a comparative analysis of the mechanical parameters obtained for the polymer material PLA and its composite, PLA+CF, in addition to the polymer material PETG and its composite, PETG+CF.

The comparison of the mechanical parameters for the 3D-printed PLA and PLA+CF specimens tested is presented in Table 14.

The insights garnered from Table 14 illuminate a distinct pattern—when subjected to the effects of mineral engine oil, the PLA material demonstrates an enhancement in its specific mechanical parameters, particularly in the time to the break and the nominal strain at the break.

In the 3D-printed PLA material samples exposed to the effect of mineral engine oil for 7 days, the average time to the break of the specimens increased by 64.15% compared to the specimens that were not exposed to the influence of mineral engine oil (Case 1). After 30 days of exposure (Case 3), the average time to the break began to decrease again, and compared to samples that were not exposed to the effect of mineral engine oil (Case 1), this value remained higher at 37.48%. This trend continued for the average nominal strain at the break as well; see Table 14. These improvements underscore the material’s capacity to withstand deformation and its resilience under the influence of the oil.

Upon closer examination, the changes observed in the tensile strengths and the Young’s modulus for the PLA material exposed to mineral engine oil are relatively subtle. These minor variations indicate that the oil’s impact on these specific parameters might be limited, suggesting that other factors or mechanisms could be at play in influencing these attributes.

In stark contrast, the scenario for the PLA+CF material reveals a more pronounced response. Notable shifts in the time to the break and the nominal strain at the break are evident, showcasing a distinct susceptibility of these properties to the influence of the mineral engine oil. This phenomenon could be attributed to the complex interaction between the oil and the composite structure, potentially leading to alterations in the material’s ability to absorb energy and deform without fracturing.

**Figure 28:**
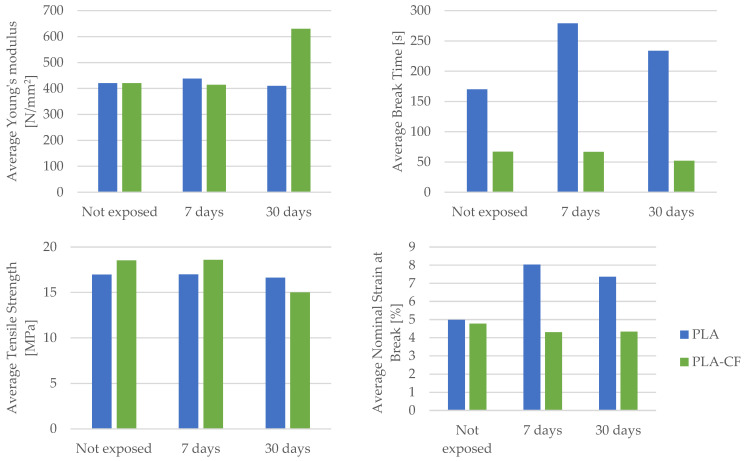
Graphical representation of the comparison of mechanical parameters between PLA and PLA+CF materials.

In essence, Figure 28 provides a comprehensive visual representation of the nuanced effects of the mineral engine oil on the PLA and PLA+CF materials. While the PLA demonstrates enhancements in select attributes, the response of the PLA+CF is marked by more substantial variations in certain mechanical parameters. This underscores the need for a detailed understanding of the intricate interactions between materials and external factors, paving the way for improved material selection and design in various applications.

The comparison of the mechanical parameters of 3D-printed PETG and PETG+CF specimens tested is presented in Table 15.

Upon dissecting the insights gleaned from Table 15, a compelling narrative unfolds. The mechanical parameters encompassing Young’s modulus, the time to the break, and nominal strain at the break exhibit a favorable transformation for both the PETG material and its composite counterpart, PETG+CF, when subjected to the influence of mineral engine oil. This phenomenon alludes to the oil’s potential to augment the material’s capacity to deform, absorb energy, and retain its structural integrity. Namely, based on the data shown in Table 15, we can observe a significant increase in the average Young’s modulus for PETG material under the influence of mineral engine oil, specifically by 55.08% after 7 days and 66.27% after 30 days.

This trend of an increasing Young’s modulus is also present in the PETG+CF material. After a 7-day exposure to mineral engine oil, the average Young’s modulus increased by 32.25%, or by 82.21% after a 30-day exposure.

Conversely, a trend emerges in terms of tensile strength. The extended exposure to mineral engine oil triggers a decline in tensile strength for both the PETG material and the PETG+CF composite. This intriguing observation could stem from various factors, including potential structural alterations or changes in the material’s ability to resist external forces due to the oil’s effects over time.

**Figure 29:**
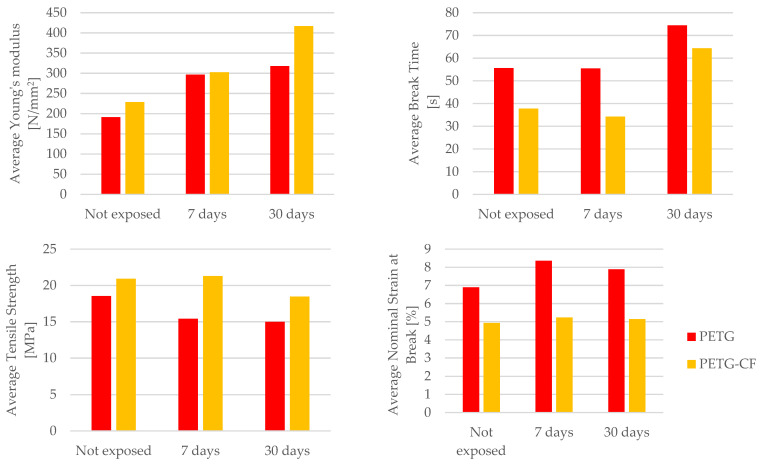
Graphical representation of the comparison of mechanical parameters between PETG and PETG+CF materials.

Figure 29 presents a graphical representation of a comparative analysis that encompasses the average Young’s modulus, the average time to break, average tensile strength, and average nominal strain at the break for both the PETG polymer material and its corresponding composite, PETG+CF.

Delving deeper into the distinction between the PETG and PETG+CF materials reveals a crucial dissimilarity in nominal strain at the break. Specifically, the response to mineral engine oil varies distinctly between the two. In the case of the PETG material, the nominal strain at the break increases in the presence of the oil (after a 7-day exposure to mineral engine oil it was 21.34%, or 14.51% after a 30-day exposure), implying an improved ability to accommodate deformation before breaking. In contrast, for the PETG+CF material, this value remains relatively stable (the nominal strain at the break was 6.08% after a 7-day exposure and 4.26% after a 30-day exposure to mineral engine oil), suggesting that the composite’s response to the oil is more regulated and less responsive to changes in the deformation behavior.

Furthermore, the passage of time imparts a unique facet to the behavior of the PETG+CF material. As the exposure to mineral engine oil persists, a characteristic rise in the time to break becomes evident during tensile testing, as vividly depicted in Figure 29. This trend highlights the evolving interaction between the composite material and the oil, potentially indicating that the composite’s energy absorption capabilities are progressively influenced by the oil’s presence.

## 4. Conclusions and Future Work

This study has provided valuable insights into the impact of mineral engine oil exposure on the mechanical parameters of 3D-printed specimens made from PLA, PLA+CF, PETG, and PETG+CF materials. These findings have significant implications for real-world applications across various industries.

For the PLA specimens, the exposure to mineral engine oil resulted in changes to the mechanical parameters. The average tensile strength experienced a reduction of approximately 36% when compared to the manufacturer-specified values for a 100% infill PLA filament. However, considering that the specimens were printed with a 30% infill, this reduction remains within acceptable limits. Furthermore, the nominal strain at the break exhibited a substantial increase (by 60.92% after 7 days and 47.49% after 30 days), indicating improved ductility. Notably, the Young’s modulus of the PLA material remained largely unaffected by exposure to the mineral engine oil.

Similarly, PLA+CF samples displayed alterations following exposure to mineral engine oil. The average tensile strength decreased by 17.56% after 30 days, while the nominal strain at the break decreased by around 10%.

In the 3D-printed PLA+CF material exposed for 30 days of operation to the mineral engine oil, there was a significant increase in the Young’s modulus (by more than 49.93%). To ensure the experiment’s repeatability, future research should involve additional tests with a larger number of samples.

For PETG specimens, the effects of the mineral engine oil were intriguing. Although the average tensile strength decreased with exposure (by 16.66% for 7 days and 16.85% for 30 days), the nominal strain at the break increased significantly (by 21.34% for 7 days and 14.51% for 30 days). This suggests that the oil enhances ductility and deformation capacity, highlighting a complex interaction between the material and the mineral engine oil. The increase in nominal strain at the break was particularly pronounced in specimens exposed to the oil for 30 days.

In 3D-printed PETG material specimens, there was a substantial increase in the Young’s modulus under the influence of mineral engine oil (by 55.08% after 7 days and 66.27% for 30 days). Additionally, there was an increase in the time to the break (by 9.14% after 7 days and 29.61% after 30 days).

PETG+CF samples exhibited similar trends, initially showing an increased tensile strength (by 1.78%) and nominal strain at the break (by 6.08%) after 7 days of exposure. However, prolonged exposure (after 30 days) led to a reduction in tensile strength (by 11.75%).

These findings hold relevance for industries where 3D-printed parts interact with fluids, such as automotive or industrial machinery. Understanding the complex interplay between materials and fluids, like mineral engine oil, can guide improved designs and material selection for components operating in such environments.

Furthermore, the study underscores the importance of conducting additional tests with larger sample sizes to ensure experimental repeatability, particularly when observing significant variations in the material parameters. This aspect is critical for industries where consistent performance and quality control are paramount.

The results suggest that, in specific scenarios, exposure to mineral engine oil can enhance the material properties, such as an increased Young’s modulus or improved ductility. These enhancements can be strategically leveraged in industries requiring specific mechanical characteristics for optimal performance.

Industries demanding long-term durability and resistance to environmental factors, such as automotive or aerospace, can use these findings to assess the performance of 3D-printed parts over extended periods of exposure to fluids like mineral engine oil.

In summary, this research provides valuable insights into the behavior of 3D-printed materials when exposed to mineral engine oil, enabling informed decisions regarding material selection, printing parameters, and the performance of 3D-printed components in practical applications. This knowledge can lead to more efficient designs, improved product durability, and enhanced performance across various industries.

The influence of mineral engine oil on the microstructure of 3D-printed polymer and composite materials is complex and can impact on their mechanical properties. Research in this direction is crucial for understanding how materials behave in real-world environments and for the development of superior materials tailored to specific applications. Taking these findings into account can contribute to enhancing the durability and efficiency of 3D-printed components in various industries. In addition, further research is recommended to gain a better understanding of these intricate interactions between materials and mineral engine oil, as well as for the development of even more advanced materials for future applications.

Future research avenues could explore a more detailed analysis of microstructural changes induced by exposure to mineral engine oil and investigate the long-term effects on material parameters and properties. Additionally, considering other environmental factors and various types of mineral engine oils could provide a more comprehensive understanding of material–oil interactions.

## Figures and Tables

**Table 1:** PLA, PLA+CF, PETG, and PETG+CF filaments specifications [68].

Material Type	PLA	PLA+CF	PETG	PETG+CF
Diameter (mm)	1.75	1.75	1.75	1.75
Color	silver	black	green	black
Net filament weight (g)	1000	1000	1000	1000
Water absorption (equilibrium in water, 23 °C)	<0.3	0.5	<0.2	0.8
Printing speed (mm/s)	40–60	60–90	60–90	60–90
Layer height (mm)	0.1–0.2	0.1–0.2	0.1–0.2	0.1–0.2
Extrusion temperature (°C)	190–220	200–230	240–250	230–250
Bed platform temperature (°C)	50–55	40–50	80–90	60–80

**Table 2:** Mechanical parameters of PLA, PLA+CF, PETG, and PETG+CF filaments [68].

Material Type	PLA	PLA+CF	PETG	PETG+CF
Density (g/cm^3^)	1.25	~1.29	1.23	~1.28
Tensile Strength (Mpa)	45–49	40–45	40–45	40–43
Flexural Strength (Mpa)	69–75	85–95	50–55	75–85
Young’s modulus (Mpa)	1000–1100	1100–1300	1000–1100	2100–2400
Elongation at Break (%)	13.5–15.5	11.5–13.5	6.0–8.0	7.5–8.5
Heat Deflection Temperature (°C)	53	60	74	70

**Table 3:** Dimensions of tensile test specimen according to ISO 527-2 in millimeters.

Dimensions	Symbol	Value
Preferred thickness	h	4.0
Length of narrow parallel-sided portion	$l_{1}$	80.0
Distance between broad parallel-sided portions	$l_{2}$	104
Overall length	$l_{3}$	150
Gauge length	$L_{0}$	75
Initial distance between grips	L	115
Width at narrow portion	$b_{1}$	10
Width at ends	$b_{2}$	20
Radius	r	24

**Table 4:** Main printing parameters.

3D Printing Parameter	PLA	PLA+CF	PETG	PETG+CF
Filament diameter (mm)	1.75	1.75	1.75	1.75
Infill pattern	Hexagon	Hexagon	Hexagon	Hexagon
Infill density (%)	30	30	30	30
Nozzle diameter (mm)	0.4	0.4	0.4	0.4
Base print speed (mm/s)	60	60	60	60
Travel speed (mm/s)	100	100	100	100
First layer maximum (mm/s)	10	10	10	10
Top solid layers	4	4	4	4
Bottom solid layers	3	3	3	3
Layer height (mm)	0.2	0.2	0.2	0.2
First layer height (mm)	0.3	0.3	0.3	0.3
Extrusion temperature (°C)	210	225	240	245
Bed temperature (°C)	50	50	90	80

**Table 5:** Recorded parameters during the 3D printing.

Recorded Parameter	PLA	PLA+CF	PETG	PETG+CF
Print time (min)	44	44	46	48
Material mass used (g)	5.91	6.82	5.94	6.23
Filament length used (m)	1.98	2.17	2.12	2.23

**Table 6:** PLA specimens tensile test results.

Case	Specimen Code	Max. Force [N]	Break Force [N]	Max. Displacement Force [N]	Max. Displacement [mm]
	PLA_211	684.079	567.063	−29.031	5.620
Case 1	PLA_212	680.113	573.349	−26.822	5.762
	PLA_213	672.595	627.049	−26.997	5.841
	Average	678.929	589.154	−27.617	5.741
	St. Dev.	5.833	32.968	1.228	0.112
	PLA_214	682.171	561.237	−28.777	8.914
Case 2	PLA_215	676.163	393.415	−25.638	9.892
	PLA_216	679.056	542.023	−28.011	8.895
	Average	679.130	498.892	27.475	9.234
	St. Dev.	3.005	91.849	1.637	0.570
	PLA_217	391.404	285.459	257.413	5.00
Case 3	PLA_218	666.078	430.147	−32.528	8.720
	PLA_219	664.314	339.707	−38.864	8.213
	Average	573.932	351.771	62.007	7.311
	St. Dev.	158.076	73.095	169.256	2.017

**Table 7:** The mechanical parameters of 3D-printed PLA specimens tested.

Case	Specimen Code	Young’s Modulus [N/mm^2^]	Break Time [s]	Tensile Strength [MPa]	Nominal Strain at Break [%]
	PLA_211	473.714	168.610	17.102	4.89
Case 1	PLA_212	453.281	169.760	17.003	5.01
	PLA_213	335.596	171.420	16.815	5.08
	Average	420.864	169.930	16.973	4.99
	St. Dev.	74.547	1.413	0.146	0.10
	PLA_214	454.095	267.390	17.054	7.75
Case 2	PLA_215	412.694	297.670	16.904	8.60
	PLA_216	446.562	271.578	16.976	7.74
	Average	437.783	278.879	16.978	8.03
	St. Dev.	22.052	16.407	0.075	0.49
	PLA_217	797.581	156.900	9.785	4.35
Case 3	PLA_218	458.760	241.001	16.652	7.58
	PLA_219	361.673	226.246	16.608	7.14
	Average	410.216	233.624	16.630	7.36
	St. Dev.	228.853	44.906	3.952	1.75

**Table 8:** PLA+CF specimens: tensile test results.

Case	Specimen Code	Max. Force [N]	Break Force [N]	Max. Displacement Force [N]	Max. Displacement [mm]
	PLA+CF_511 *	726.247	-	−26.083	5.758
Case 4	PLA+CF_512 *	765.363	-	−19.399	5.821
	PLA+CF_513	731.961	693.274	−26.878	4.898
	Average	741.190	693.274	−24.120	5.492
	St. Dev.	21.128	0	4.108	0.516
	PLA+CF_514	721.073	615.279	−34.444	4.614
Case 5	PLA+CF_515	728.693	621.982	−33.257	4.724
	PLA+CF_516	782.283	735.370	−23.969	5.486
	Average	744.016	657.544	−30.557	4.941
	St. Dev.	33.358	67.483	5.736	0.475
	PLA+CF_517 *	643.627	-	175.436	5.328
Case 6	PLA+CF_518	636.911	398.421	−87.357	5.469
	PLA+CF_519	552.630	288.681	−174.720	4.125
	Average	611.056	342.051	−145.838	4.974
	St. Dev.	50.710	77.598	182.255	0.739

* Error on the specimen—the data are not included in further calculations.

**Table 9:** The mechanical parameters of 3D-printed PLA+CF specimens tested.

Case	Specimen Code	Young’s Modulus [N/mm^2^]	Break Time [s]	Tensile Strength [MPa]	Nominal Strain at Break [%]
	PLA+CF_511	496.449	65.1300	18.156	5.01
Case 4	PLA+CF_512	347.645	69.0700	19.134	5.06
	PLA+CF_513	417.481	67.1800	18.299	4.26
	Average	420.525	67.1267	18.530	4.78
	St. Dev.	74.449	1.971	0.528	0.45
	PLA+CF_514	405.056	59.9300	18.027	4.01
Case 5	PLA+CF_515	428.230	67.0700	18.217	4.11
	PLA+CF_516	409.954	73.0100	19.557	4.77
	Average	414.413	66.6700	18.600	4.30
	St. Dev.	12.214	6.549	0.834	0.41
	PLA+CF_517	517.950	50.2600	16.091	4.63
Case 6	PLA+CF_518	342.150	61.2300	15.923	4.76
	PLA+CF_519	1031.37	44.9200	13.816	3.59
	Average	630.490	52.1367	15.277	4.33
	St. Dev.	358.127	8.315	1.268	0.64

**Table 10:** PETG specimens tensile test results.

Case	Specimen Code	Max. Force [N]	Break Force [N]	Max. Displacement Force [N]	Max. Displacement [mm]
	PETG_411	760.643	575.288	−2.901	8.423
Case 7	PETG_412 *	753.291	-	42.733	9.916
	PETG_413	711.131	531.840	−26.417	5.441
	Average	741.688	553.564	4.471	7.926
	St. Dev.	26.718	30.722	35.160	2.278
	PETG_414	591.532	-	317.327	10.321
Case 8	PETG_415	589.760	-	187.524	10.096
	PETG_416	668.836	-	210.412	8.414
	Average	616.709	-	238.421	9.610
	St. Dev.	45.152	0	69.286	1.042
	PETG_417	627.780	-	213.782	10.911
Case 9	PETG_418	610.693	-	283.877	7.794
	PETG_419	615.803	-	511.758	8.511
	Average	618.092	-	336.472	9.072
	St. Dev.	8.770	0	155.795	1.632

* Error on the specimen—the data are not included in further calculations.

**Table 11:** Mechanical parameters of 3D-printed PETG specimens tested.

Case	Specimen Code	Young’s Modulus [N/mm^2^]	Break Time [s]	Tensile Strength [MPa]	Nominal Strain at Break [%]
	PETG_411	186.386	77.3800	19.016	7.32
Case 7	PETG_412 *	-	41.3700	18.832	8.62
	PETG_413	196.510	48.1700	17.778	4.73
	Average	191.448	55.6400	18.542	6.89
	St. Dev.	7.159	19.132	0.668	1.98
	PETG_414	500.963	49.1000	14.788	8.98
Case 8	PETG_415	100.086	68.0000	14.744	8.78
	PETG_416	289.632	49.2300	16.721	7.32
	Average	296.894	55.4330	15.418	8.36
	St. Dev.	200.537	10.875	1.129	0.91
	PETG_417	324.597	71.5800	15.695	9.49
Case 9	PETG_418	305.941	71.3900	15.267	6.78
	PETG_419	324.430	71.2300	15.395	7.40
	Average	318.323	74.4000	15.452	7.89
	St. Dev.	10.723	0.175	0.220	1.42

* Error on the specimen—the data are not included in further calculations.

**Table 12:** PETG+CF specimens: tensile test results.

Case	Specimen Code	Max. Force [N]	Break Force [N]	Max. Displacement Force [N]	Max. Displacement [mm]
	PETG+CF_711	826.375	726.660	−19.407	5.238
Case 10	PETG+CF_712	879.439	577.633	3.568	5.387
	PETG+CF_713	805.489	521.827	−18.009	6.387
	Average	837.101	608.707	−11.183	5.670
	St. Dev.	38.124	105.893	12.880	0.625
	PETG+CF_714	923.840	505.575	−3.171	5.256
Case 11	PETG+CF_715	829.419	426.865	−25.900	5.401
	PETG+CF_716	802.557	-	−17.452	7.368
	Average	851.939	455.220	−15.508	6.008
	St. Dev.	63.700	55.656	11.489	1.180
	PETG+CF_717	763.973	441.400	−24.255	6.386
Case 12	PETG+CF_718	755.556	425.792	−16.022	5.259
	PETG+CF_719	696.675	-	−84.536	6.096
	Average	738.735	433.596	−41.604	5.914
	St. Dev.	36.667	11.037	37.407	0.585

**Table 13:** Mechanical parameters of 3D-printed PETG+CF specimens tested.

Case	Specimen Code	Young’s Modulus [N/mm^2^]	Break Time [s]	Tensile Strength [MPa]	Nominal Strain at Break [%]
	PETG+CF_711	327.756	35.1600	20.659	4.55
Case 10	PETG+CF_712	188.796	35.1600	21.986	4.68
	PETG+CF_713	169.598	43.0000	20.137	5.55
	Average	228.717	37.7733	20.927	4.93
	St. Dev.	86.306	4.526	0.953	0.54
	PETG+CF_714	264.398	32.9100	23.096	4.57
Case 11	PETG+CF_715	366.040	35.0300	20.736	4.70
	PETG+CF_716	277.015	34.7200	20.064	6.41
	Average	302.484	34.2200	21.299	5.23
	St. Dev.	55.401	1.145	1.592	1.03
	PETG+CF_717	355.564	70.1600	19.099	5.55
Case 12	PETG+CF_718	321.530	63.7400	18.889	4.57
	PETG+CF_719	573.109	59.1900	17.417	5.30
	Average	416.734	64.3633	18.468	5.14
	St. Dev.	136.489	5.512	0.917	0.51

**Table 14:** Comparison of mechanical parameters of 3D-printed PLA and PLA+CF specimens tested.

Material	Case	Average Young’s Modulus (St. Dev.) [N/mm^2^]	Average Break Time (St. Dev.) [s]	Average Tensile Strength (St. Dev.) [MPa]	Average Nominal Strain at Break (St. Dev.) [%]
PLA	Not exposed	420.864 (74.547)	169.930 (1.413)	16.973 (0.146)	4.99 (0.10)
	7 days	437.783 (22.052)	278.879 (16.407)	16.978 (0.075)	8.03 (0.49)
	30 days	410.216 (228.853)	233.624 (44.906)	16.630 (3.952)	7.36 (1.75)
PLA+CF	Not exposed	420.525 (74.449)	67.127 (1.971)	18.530 (0.528)	4.78 (0.45)
	7 days	414.413 (12.214)	66.670 (6.549)	18.600 (0.834)	4.30 (0.41)
	30 days	630.490 (358.127)	52.137 (8.315)	15.277 (1.268)	4.33 (0.64)

**Table 15:** Comparison of mechanical parameters of 3D-printed PETG and PETG+CF specimens tested.

Material	Case	Average Young’s Modulus (St. Dev.) [N/mm^2^]	Average Break Time (St. Dev.) [s]	Average Tensile Strength (St. Dev.) [MPa]	Average Nominal Strain at Break (St. Dev.) [%]
PETG	Not exposed	191.448 (7.159)	37.773 (19.132)	18.542 (0.668)	6.89 (1.98)
	7 days	296.894 (200.537)	34.220 (10.875)	15.418 (1.129)	8.36 (0.91)
	30 days	318.323 (10.723)	64.363 (0.175)	15.452 (0.220)	7.89 (1.42)
PETG+CF	Not exposed	228.717 (86.306)	37.773 (4.526)	20.927 (0.953)	4.93 (0.54)
	7 days	302.484 (55.401)	34.220 (1.145)	21.299 (1.592)	5.23 (1.03)
	30 days	416.734 (136.489)	64.363 (5.512)	18.468 (0.917)	5.14 (0.51)

## Data Availability

Data sharing available upon request.
